# Prediction of the disease burden of nasopharyngeal carcinoma in China: A comparative analysis of ARIMA, LSTM, and DNN models

**DOI:** 10.1097/MD.0000000000046037

**Published:** 2025-11-21

**Authors:** Yun Chen, Kai Zhang, Jian Luo

**Affiliations:** a Otolaryngology Head and Neck surgery, Yibin First People’s Hospital, Yibin, Sichuan, People’s Republic of China.

**Keywords:** disease burden, machine learning, nasopharyngeal carcinoma, prediction

## Abstract

Nasopharyngeal carcinoma (NPC), highly prevalent in southern China, often presents insidious early symptoms, resulting in advanced-stage diagnosis, significant treatment challenges, and poor prognosis. Accurate prediction of NPC’s disease burden is essential for devising effective prevention and treatment strategies and optimizing medical resource allocation. This study used the 2021 Global Burden of Disease (GBD) study’s disability-adjusted life years (DALYs) data on NPC in China. It developed 3 disease burden prediction models (ARIMA, deep neural networks [DNN], and long short-term memory [LSTM]), whose performance was assessed by mean absolute error, mean absolute percentage error, and RMSE. The DNN model demonstrated superior fitting on the training set with the lowest error metrics, yet it exhibited overfitting as its performance declined on the testing set. In contrast, the ARIMA model, with its assumption of stationarity, achieved the best generalization with the lowest mean absolute error and mean absolute percentage error on the testing data. The LSTM model recorded higher errors on the test set. Forecasts for 2022 to 2030 showed that while ARIMA predicted stable DALYs, both DNN and LSTM models indicated a gradual decline. The future predictions of all 3 models indicate that although the disease burden of NPC may gradually decrease, it remains extremely severe. This study compared ARIMA, DNN, and LSTM models for predicting NPC disease burden in China. Although DNN excelled on training data, ARIMA generalized best on testing, while LSTM struggled due to limited data. Future research should integrate diverse sources and improve interpretability to support NPC prevention and treatment.

## 1. Introduction

Nasopharyngeal carcinoma (NPC), a malignant tumor originating from the nasopharyngeal mucosal epithelium, exhibits unique epidemiological characteristics and clinical manifestations. Globally, NPC incidence shows significant geographical and racial disparities, with a particularly high prevalence in southern China.^[1,2]^ The insidious nature of early symptoms frequently results in late diagnosis for most patients, complicating treatment and contributing to a poor prognosis. Although medical advancements have improved NPC diagnosis and treatment, the disease’s high incidence and mortality rates remain significant challenges to public health. Consequently, thorough investigation into NPC disease burden prediction models is crucial for developing effective prevention and treatment strategies and for optimizing medical resource allocation.^[3,4]^

Disease burden prediction, a key aspect of public health research, involves scientifically forecasting the incidence, medical resource needs, and economic impacts of specific diseases over future time frames through mathematical modeling and statistical analysis. Accurate predictions are essential for guiding government health policy development, resource allocation, and disease prevention and control efforts. In the complex context of nasopharyngeal carcinoma, research on disease burden prediction is still in its infancy.^[5–7]^ Traditional prediction methods, reliant on historical data trends and expert intuition, inherently possess subjectivity and uncertainty. The advent of big data and artificial intelligence technologies has prompted a growing number of scholars to employ sophisticated mathematical models and machine learning algorithms in disease burden prediction, thereby seeking to improve accuracy and reliability.^[8,9]^

Time series prediction techniques are pivotal for analyzing historical data to forecast future trends, with broad applications in economics, medicine, and engineering.^[10–12]^ Traditional models, such as the autoregressive integrated moving average (ARIMA), facilitate short-term predictions by capturing data autocorrelation, trends, and seasonality.^[13–15]^ While these statistically grounded models offer clear mathematical structures and robust interpretability, they may exhibit limitations in managing complex nonlinear relationships and large datasets.

The swift progress of artificial intelligence has introduced deep learning technologies as innovative tools for time series prediction, with deep neural networks (DNN) and long short-term memory networks (LSTM) being prominent examples. DNNs, leveraging multiple layers of nonlinear transformations, excel in learning complex data features and patterns, thereby exhibiting robust nonlinear modeling capabilities.^[16–18]^ LSTM, a refined variant of recurrent neural networks, is tailored for time series data processing and prediction. Its gating mechanisms mitigate the gradient vanishing issue inherent in recurrent neural networks, facilitating stable information flow across extended time periods.^[19–21]^ The strength of deep learning in time series prediction is rooted in its superior feature extraction and modeling abilities, making it well-suited for high-dimensional, nonlinear, and dynamically complex data. Nevertheless, these models often necessitate substantial data for training and exhibit intricate architectures, which can compromise interpretability. This study undertakes a comparative analysis of the ARIMA, DNN, and LSTM models’ performance in predicting the disease burden of NPC, delineating the strengths, weaknesses, and suitable contexts of each model. The findings aim to provide scientific rationale and decision-making support for the advancement of NPC prevention and treatment strategies.

## 2. Materials and methods

This study complies with the GATHER guidelines.^[22]^

### 2.1. Data source

This study used open data from the 2021 Global Burden of Disease (GBD)(https://vizhub.healthdata.org/gbd-results/)^[23]^ study to assess the disease burden of nasopharyngeal carcinoma in China, therefore there is no ethical approval statement for this study. The GBD adopted a set of interrelated indicators to determine the incidence rate, prevalence rate, mortality rate, years of lost life and disability-adjusted life years (DALYs) of 371 diseases in 204 countries and regions, and its method is detailed in the literature.

DALYs are of great significance when assessing the burden of disease. DALYs comprehensively considers the deaths and disabilities caused by diseases and can fully reflect the impact of diseases on the health of the population. By combining mortality and morbidity, DALYs can reveal the overall burden of the disease, not just the impact caused by death. Furthermore, DALYs can be compared across diseases, providing burden assessment among different diseases, which is conducive to the formulation of public health policies and the allocation of resources. In this study, the use of DALYs can more accurately quantify the disease burden of NPC, providing a scientific basis for optimizing prevention and treatment strategies.

Data integration in the GBD study spans diverse sources, including civil registration, vital statistics, household surveys, and hospital records, published research, clinical documentation, census data, surveillance records, health service utilization, insurance claims, and other relevant datasets. Among the myriad causes of death or injury studied, such as neoplasms and cardiovascular diseases, this study concentrated on NPC, defined by the International Classification of Diseases code ICD-10 (C11). We extracted DALYs data for nasopharyngeal carcinoma in China from 1990 to 2021 from the GBD database.

### 2.2. Data preprocessing

Before the model was developed, the original data was preprocessed to ensure its quality and the applicability of time series predictions. The original data of GBD is very complete, with no missing values or outliers. To ensure consistent proportions and improve model performance, MinMaxScaler was used to normalize the features, which scaled the data to a range between 0 and 1. This step is particularly important for neural network-based models, including DNN and LSTM, which are very sensitive to the size of the input features. Finally, the dataset was divided into training and test subsets. Among them, 80% of the data was used for training (1990–2015), and the remaining 20% was used for model evaluation (2016–2021). These preprocessing processes ensure that the data is clean, balanced, and ready for predictive modeling.

### 2.3. ARIMA model

The ARIMA model is an important tool for time series forecasting, capable of capturing autocorrelation, trends, and seasonal variations in the data.^[24]^ The construction process begins with the application of the Augmented Dickey-Fuller (ADF) test to assess data stationarity; for non-stationary series, differencing is used to determine an appropriate d value. Hyperparameter tuning involves a grid search for the optimal combination of p, d, and q parameters, typically ranging from 0 to 5 for p and q, while d is set to 0 or 1 based on stationarity. Information criteria such as AIC and Bayesian information criterion are used to select the best model. The performance is further validated through residual analysis and white noise tests, ensuring that the residuals are uncorrelated and exhibit no autocorrelation.

### 2.4. DNN model

The DNN model, with its multiple hidden layers and nonlinear activation functions, is sensitive to overfitting, especially due to its high complexity.^[25]^ Hyperparameters such as the number of hidden layers and units per layer, activation functions, learning rate, batch size, and dropout rate are optimized using grid search or random search techniques. The dropout rate is typically set between 0.2 and 0.5 to prevent overfitting, while the learning rate is tuned with values ranging from 0.001 to 0.01. Regularization techniques like early stopping and the use of the Adam optimizer are employed to enhance generalization. The final architecture is selected based on the model’s ability to generalize well to unseen data while minimizing overfitting.

### 2.5. LSTM model

**Figure 1.**
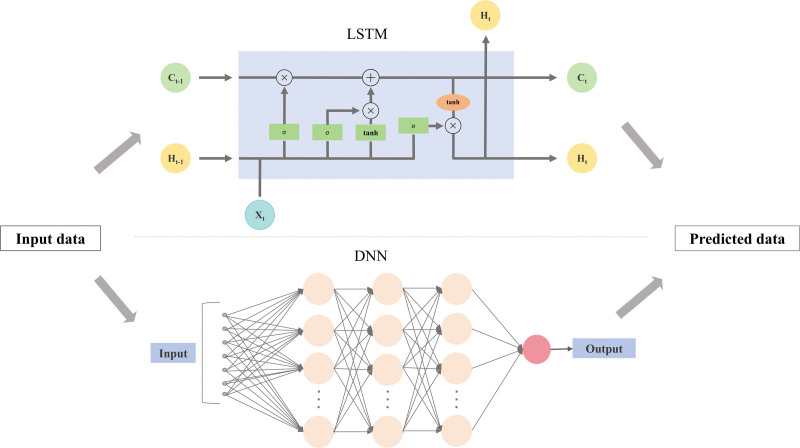
Schematic diagram of the deep neural network (DNN) and long short-term memory (LSTM) network models.

LSTM models, designed to handle long-term dependencies in sequential data, require careful tuning of hyperparameters, including the number of layers, units per layer, dropout rate, learning rate, and sequence length.^[26]^ Typically, the number of layers is set between 1 and 3, with units per layer varying from 50 to 200. Dropout rates between 0.2 and 0.5 are tested to avoid overfitting, and the learning rate is adjusted in the range of 0.001 to 0.01. The sequence length is optimized depending on the time series data, generally tested for values between 10 and 50 time steps. Hyperparameter optimization is done through grid search or random search, with validation data used to select the best-performing model based on forecasting accuracy. The schematic diagram of the DNN and LSTM network models is shown in Figure 1. The hyperparameter Settings of ARIMA, DNN and LSTM are shown in Table 1.

**Table 1:** The hyperparameter settings of ARIMA, DNN, and LSTM.

Model	Hyperparameter	Value
ARIMA	Autoregressive order (*p*)	2
	Degree of differencing (*d*)	0
	Moving average order (*q*)	0
	Selection criterion	BIC minimization (selected ARIMA(2,0,0))
	Train/test split	80%/20%
DNN	Number of hidden layers	2
	Units per hidden layer	64
	Activation function	ReLU
	Optimizer	Adam
	Learning rate	0.001
	Batch size	4
	Epochs	200
	Loss function	Mean squared error (MSE)
	Train/test split	80%/20%
LSTM	Look-back window (sequence length)	3 (using past 3 years to predict the next year)
	Number of LSTM layers	1
	Hidden units per layer	50
	Optimizer	Adam
	Learning rate	0.001
	Batch size	1
	Epochs	200
	Loss function	Mean squared error (MSE)
	Train/Test split	80%/ 20%

ARIMA = autoregressive integrated moving average, BIC = Bayesian information criterion, DNN = deep neural network, LSTM = long short-term memory.

### 2.6. Model comparison methods

In model evaluation, 3 key performance metrics – root mean square error (RMSE), mean absolute error (MAE), and mean absolute percentage error (MAPE) – are essential for assessing and comparing the fitting and predictive accuracy of 3 distinct models. Lower values in these metrics indicate superior predictive performance. The MAE, a basic measure of predictive accuracy, quantifies the average difference between predicted and actual values. The MAPE, which averages unsigned percentage errors, effectively distinguishes between error magnitudes but may underestimate the impact of rare errors. In contrast, the RMSE, due to its sensitivity to outliers, amplifies prediction errors, providing a detailed and accurate assessment of predictive performance. The computational formulas for these metrics are presented in Table 2.

**Table 2:** Model evaluation metrics and their calculation formulas.

Evaluation indicators	Meaning	Calculation formula	Remarks
Mean squared error, MSE	The average of the squared differences between the predicted values and the actual values	$MSE=\frac{1}{n}\overset{n}{\underset{i=1}{\sum }}{\left(y_{i}-{\hat{y}}_{i}\right)}^{2}$	n is the number of samples, $y_{i}$ is the actual value, and ${\hat{y}}_{i}$ is the predicted value.
Mean absolute error, MAE	The average of the absolute differences between the predicted values and the actual values	$MAE=\frac{1}{n}\underset{i=1}{\overset{n}{\sum }}\;y_{i}-{\hat{y}}_{i}$	
Mean absolute percentage error, MAPE	The mean of the percentages of the absolute errors between the predicted value and actual values relative to the actual values	$MAPE=\frac{1}{n}\underset{i=1}{\overset{n}{\sum }}\;\frac{y_{i}-{\hat{y}}_{i}}{y_{i}}\times 100\%$	

### 2.7. Data grouping and tools

The training set contains data from 1990 to 2015, while the test set covers data from 2016 to 2021. Both are used to build 3 forecasting models: ARIMA, LSTM, and DNN. All model construction and data analysis were completed using Python 3.12.6 (Python Software Foundation, Wilmington).

## 3. Results

### 3.1. Descriptive statistics

**Figure 2.**
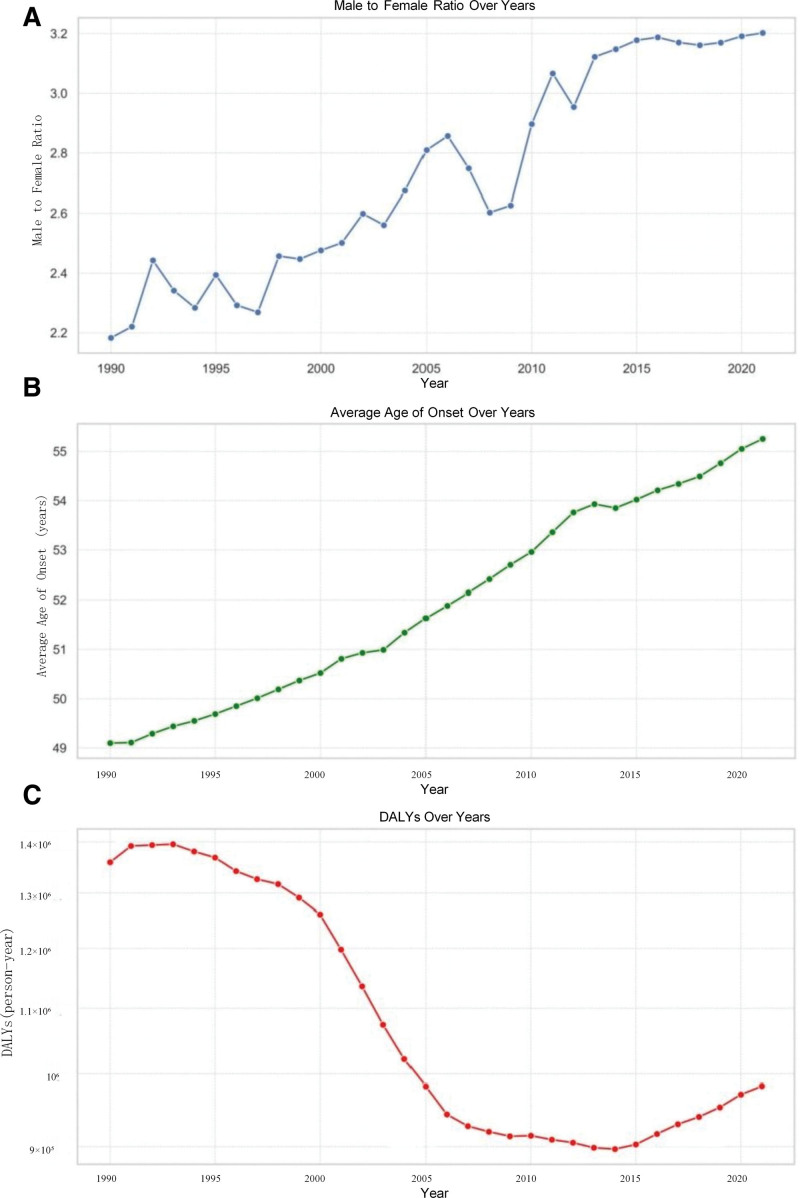
Temporal trends in the gender ratio, mean age of onset, and DALYs among patients with NPC in China from 1990 to 2021. (A) temporal trends of patient gender ratio; (B) temporal trends of mean age of onset; (C) temporal trends of DALYs among. DALYs = disability-adjusted life years, NPC = nasopharyngeal carcinoma.

The results are shown in Figure 2, which illustrates the trends in the gender ratio, average age at onset, and DALYs of NPC patients in China from 1990 to 2021. Since the age groups in the GBD database are represented as age ranges, we used the midpoint of each group for description. For example, we used “32 years” as the midpoint for the “30 to 34 years” group. The figure shows that the male-to-female ratio fluctuated with an increasing trend over time, with the proportion of male patients gradually rising. The average age at onset has remained relatively stable but steadily increased each year. DALYs reached the lowest point in 2014 before rebounding. During the entire study period, nasopharyngeal carcinoma in China contributed a total of 35,062,150.41 DALYs, with an average age at onset of 51.62 ± 14.74 years and a male-to-female ratio of 2.6396.

**Figure 3.**
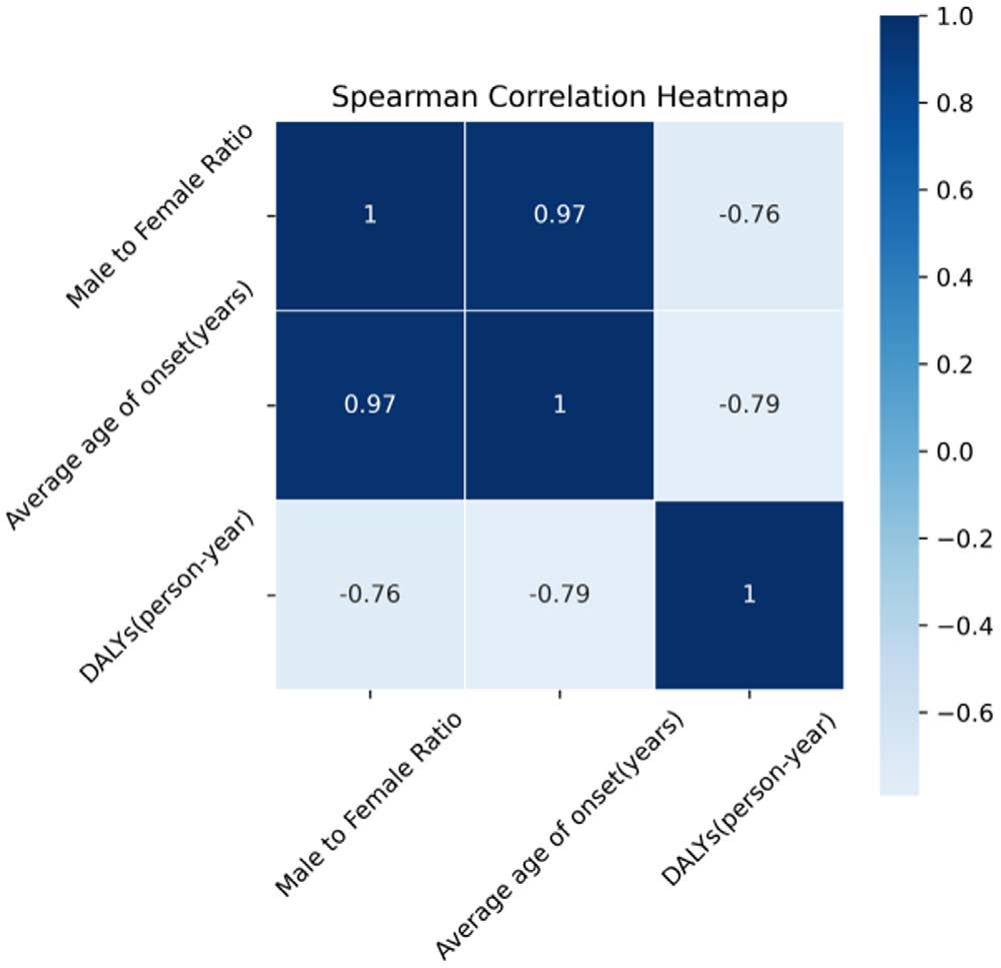
Heatmap of univariate spearman correlation analysis between DALYs for NPC and gender ratio, mean age of onset. DALYs = disability-adjusted life years, NPC = nasopharyngeal carcinoma.

Univariate Spearman correlation analysis showed that the DALYs of nasopharyngeal carcinoma were significantly negatively correlated with both the male-to-female ratio and the average age of onset, with correlation coefficients of −0.7587 and −0.7911, respectively (Fig. 3).

### 3.2. ARIMA model

#### 3.2.1. Testing for stationarity

**Figure 4.**
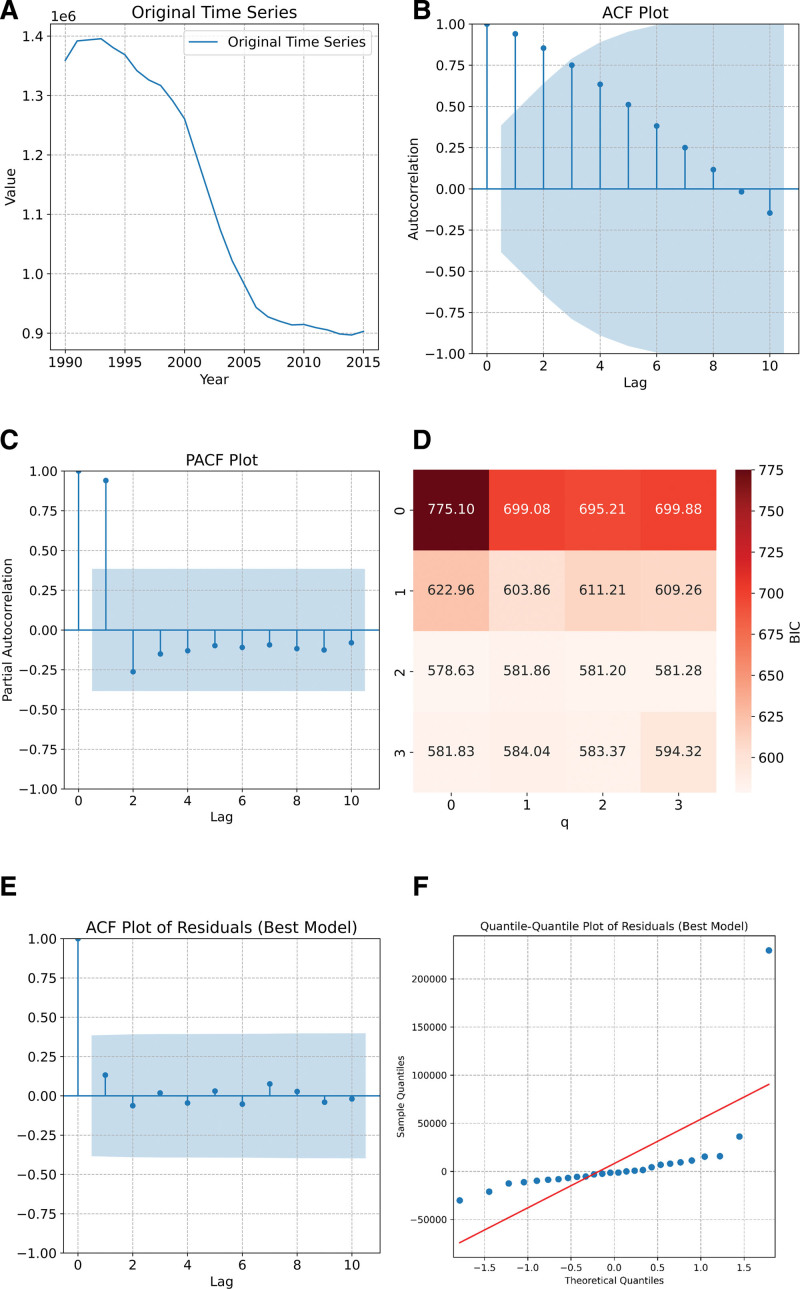
(A) ARIMA model of the DALY time series for NPC in China, 1990–2015; (B) autocorrelation function (ACF) plot; (C) partial autocorrelation function (PACF) plot; (D) BIC values for ARIMA models with different *p* and *q* values; (E) ACF plot of residuals (best model); (F) quantile-quantile plot of residuals (best model). ACF = autocorrelation function, ARIMA = autoregressive integrated moving average, DALYs = disability-adjusted life years, NPC = nasopharyngeal carcinoma.

The training set’ s original sequence exhibited a stationary trend (Fig. 4A). The Augmented Dickey-Fuller (ADF) test yielded a *t*-value of −3.586 (*P* = .006), rejecting the null hypothesis and confirming the sequence’ s stationarity. Consequently, no differencing was applied, and the parameter (d) was set to 0.

#### 3.2.2. Model identification and selection

The autocorrelation function (ACF) (Fig. 4B) and partial autocorrelation function (PACF) (Fig. 4C) plots of the time series indicated that the ACF dropped rapidly after the first few lags and remained below the confidence interval (typically ± 2 standard errors) for most lags, suggesting the absence of a tailing effect since the autocorrelation coefficients approached zero after a few lags. The PACF decayed rapidly after the first lag and fluctuated slightly in the negative direction along the zero axis, mostly within the confidence interval. As shown in Figure 4D, the Bayesian information criterion (BIC) was minimized at a value of 578.63 when (*p*) was 2 and (*q*) was 0, thereby indicating that the optimal model was ARIMA(2,0,0).

#### 3.2.3. Model testing and forecasting

The ACF plot of the residuals (Fig. 4E) shows all points within the confidence bands, suggesting no significant autocorrelation. Additionally, the Q-Q plot (Fig. 4F) indicates that the residuals are normally distributed. These findings validate the ARIMA(2,0,0) model as both valid and satisfactory, fulfilling the necessary criteria.

### 3.3. LSTM model

First, the data is loaded and extracted, then converted into floating-point arrays. To enhance the model’s training performance, the original data is normalized to a 0 to 1 range using the MinMaxScaler, ensuring consistent scaling across the dataset. Next, a supervised learning dataset is constructed by setting the look-back window to 3, which generates the corresponding input sequences and target outputs. The data is then split into training and test sets as planned, and the input data is reshaped into the 3-dimensional format (samples, timesteps, features) required by the LSTM model.

**Figure 5.**
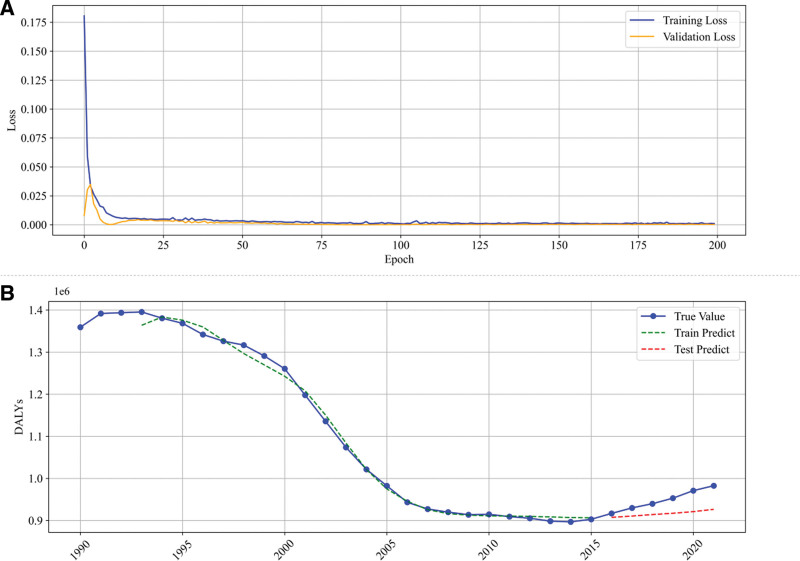
(A) LSTM loss function curve; (B) comparison between actual and LSTM predicted curves. LSTM = long short-term memory.

The model is built using Keras Sequential model. Initially, an LSTM layer with 50 units is added as the feature extraction layer, followed by a fully connected (Dense) layer with a single neuron to output the predictions. During compilation, the Adam optimizer is used along with MSE as the loss function. The model is trained for 200 epochs with a batch size of 1, and 10% of the training data is reserved as a validation set to evaluate performance. After training, predictions are made on both the training and test sets, and the scaler.inverse_transform function is employed to revert the normalized predictions back to their original scale. Additionally, the Matplotlib library is used to visualize both the trends of training and test losses and the prediction curves for the training and test sets (Fig. 5).

### 3.4. DNN model

In this study, we developed a DNN model using the Keras framework. To improve model convergence efficiency, we applied the MinMaxScaler to normalize the data within the range of 0 to 1. Next, to capture the characteristics of the time series, we customized a sliding window function called create_dataset, which uses the values of the past 3 years as input features to predict the DALYs of the next year. This approach constructs a time series dataset suitable for supervised learning. The dataset was chronologically divided into training and test sets, with 80% allocated to the training set and 20% to the test set.

**Figure 6.**
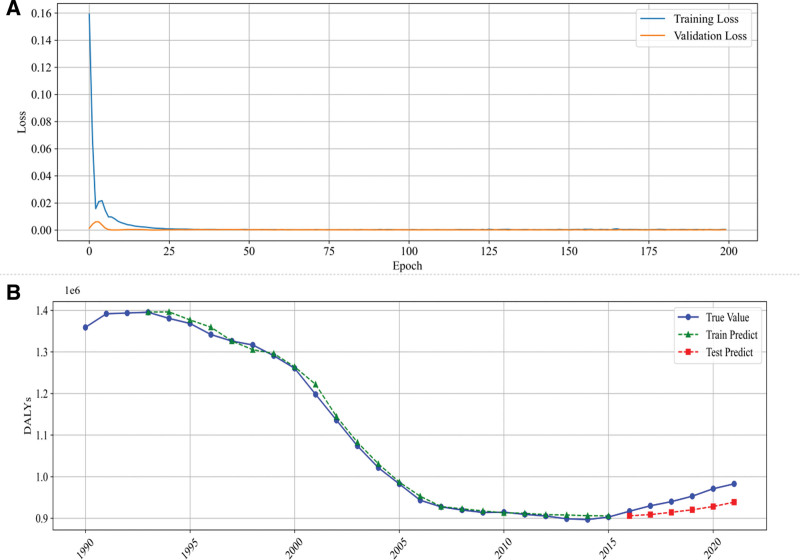
(A) DNN loss function curve; (B) comparison between true and DNN predicted curves. DNN = deep neural network.

For model construction, we designed a DNN based on Keras Sequential model. The architecture includes 2 hidden layers, each containing 64 neurons with the ReLU activation function to enhance nonlinear mapping capabilities; finally, a single-neuron output layer predicts the DALYs values. During model compilation, we selected the Adam optimizer and used MSE as the primary loss function. The model was trained for 200 epochs with a batch size of 4, while 10% of the training data was set aside as a validation set to monitor performance. After training, predictions were made on both the training and test sets, and the scaler.inverse_transform function was used to restore the normalized prediction results to their original scale. The prediction performance was then evaluated using RMSE. Finally, with the help of the Matplotlib library, we plotted comparative graphs showing the actual data alongside the training and test predictions over different years, as well as the curves of training loss and validation loss during the training process, which visually demonstrated the model’s fitting performance and convergence trend (Fig. 6).

### 3.5. Comparison of performance of different models

The prediction results of the 3 models (ARIMA, LSTM, and DNN) on both the training and test sets, along with a comparison of their performance using MAE, MAPE, and RMSE, are summarized in Table 3. It can be observed that on the training set, the DNN model has significantly lower MAE, MAPE, and RMSE than the other 2 methods, indicating a better fit to the training data. The LSTM model ranks second, while the ARIMA model shows the highest errors during training. Notably, on the test set, the ARIMA model achieves the lowest MAE and MAPE, demonstrating stronger generalization capabilities. The DNN model’s errors lie between those of ARIMA and LSTM, whereas the LSTM model exhibits the highest error metrics, indicating relatively weaker performance. Overall, although the DNN model performs best on the training set, it falls behind ARIMA on the test set, and the LSTM model experiences larger errors in the testing phase, making it difficult to surpass the other 2 models.

**Table 3:** Performance comparison of different models.

Model	Training			Testing		
	MAE	MAPE	RMSE	MAE	MAPE	RMSE
ARIMA	111,519.84	8.24%	375,575.65	24,146.95	2.52%	27,040.29
LSTM	8587.39	0.73%	12,334.07	34,367.81	3.58%	37,950.64
DNN	5760.79	0.51%	8144.84	27,988.91	2.92%	30,447.67

ARIMA = autoregressive integrated moving average, DNN = deep neural network, LSTM = long short-term memory, MAE = mean absolute error, MAPE = mean absolute percentage error, RMSE = root mean square error.

### 3.6. Prediction of future trends by 3 models

**Figure 7.**
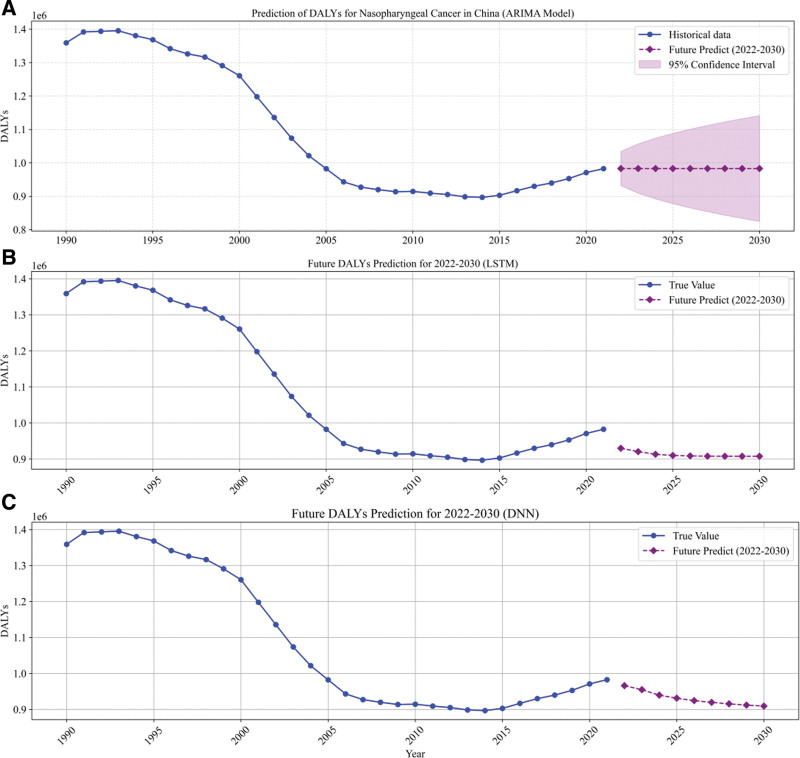
Forecasted change curves for 2022–2030 by 3 models. (A) ARIMA model (95% CI: 1.06–1.23); (B) LSTM model (95% CI: 1.03–1.35); (C): DNN model (95% CI: 1.23–1.52). ARIMA = autoregressive integrated moving average, DNN = deep neural network, LSTM = long short-term memory.

In this study, we developed ARIMA, LSTM, and DNN models to forecast the future trends of DALYs for NPC in China. The primary objective was to understand the potential evolution of the disease burden. After constructing and validating the models using historical data, we applied each model to predict the DALYs data for the 9-year period from 2022 to 2030. The forecasted results were visualized together with the historical observations, as shown in Figure 7. This combined visualization approach facilitates a direct comparison of the predictive performance of different methods. Analysis of Figure 7 reveals significant differences among the forecasts produced by the 3 models. The ARIMA model, a classical statistical forecasting method renowned for its efficiency in modeling time series data with stable patterns, predicts that the DALYs levels will remain essentially unchanged throughout the forecast period, maintaining a level nearly identical to that of 2021. This outcome indicates that, according to the ARIMA model, the underlying factors influencing the disease burden may not undergo significant changes in the coming years. In contrast, the deep learning-based LSTM and DNN models forecast a gradual decline in DALYs over the 9-year period. Both models predict that by 2030, the DALYs could decrease to approximately 90% of the 2021 level. This prediction suggests that deep learning models are capable of capturing complex nonlinear relationships and long-term dependencies in the data, potentially reflecting the cumulative impact of improvements in healthcare practices, enhanced early detection, or optimized treatment strategies. These markedly different forecast results underscore the importance of selecting an appropriate model for predictive tasks. While the ARIMA model offers a conservative outlook based on historical stability, the LSTM and DNN models demonstrate a more dynamic trend by identifying intricate patterns in the data.

## 4. Discussion

This study systematically evaluated the performance of ARIMA, DNN, and LSTM models in predicting the disease burden of NPC in China. The results indicated that the DNN model performed exceptionally well on the training set, with error metrics such as MAE, MAPE, and RMSE significantly lower than those of the ARIMA and LSTM models. This clearly demonstrates the model’s strong capability in capturing the intrinsic characteristics of the training data. However, on the test set, the ARIMA model – relying on the assumption of time series stationarity – exhibited more robust generalization performance, with prediction errors considerably lower than those of the DNN model, thereby indicating a promising prospect for practical applications. In contrast, the LSTM model, due to limitations in the data characteristics, showed relatively large prediction errors during the testing phase and failed to surpass the performance of the other models.

Further analysis revealed that although the DNN model was able to thoroughly learn the complex features present in the sample during training, its tendency to overfit resulted in poorer adaptability when faced with unseen data, leading to unsatisfactory performance on the test set. In comparison, the ARIMA model, by delving into the trends and cyclical fluctuations of historical data, maintained a high level of predictive accuracy in scenarios where data variations were not drastic.^[27–29]^ Although LSTM models theoretically have the advantage of capturing long-term dependencies, in this study, the limited data volume and relatively simplistic feature set prevented this advantage from being fully realized.^[30–32]^ Moreover, the differences in parameter tuning, training speed, and complexity among the models offer valuable insights into how to balance fitting capability and generalization performance in future practical applications.

The 3 models (ARIMA, LSTM, and DNN) produced significantly different forecasts for the future trends of DALYs in NPC. While the ARIMA model predicts stable DALYs levels, the LSTM and DNN models forecast a gradual decline, suggesting the impact of advancements in healthcare and treatment strategies. These results highlight the importance of model selection and demonstrate the varying perspectives provided by statistical and deep learning approaches.

In parallel, within the realm of machine learning for time series analysis, researchers have proposed a time series machine learning framework for the short-term and long-term forecasting of COVID-19 cases utilizing wastewater-based epidemiology data. This framework is delineated by 4 pivotal stages: feature engineering, feature selection, algorithmic tuning, and performance assessment. Empirical validation has substantiated the efficacy of this approach in predicting COVID-19 cases. The research has further revealed that time series machine learning methodologies surpass conventional forecasting techniques, particularly in the context of high-entropy data, and that the process of feature selection is paramount in enhancing the model’ s predictive accuracy and interpretability.^[33]^

Other investigators have explored the application of automated time series analysis (AutoTS) techniques to forecast hospital admissions data for the 10 leading causes of death in Romania over the period from 2008 to 2018. Leveraging the AutoTS platform, they conducted an analysis of time series data pertaining to hospital admissions, identifying the most precise prediction models to forecast monthly new cases for each NUTS 2 region for the years 2019 and 2020. The research demonstrated the efficacy of the AutoTS platform in both time series prediction and the selection of optimal models. The prediction results suggested a general trend of stability or slight decline in hospital admissions for most diseases over the subsequent 2 years, with notable variations observed across different regions and diseases.^[34]^

This study is subject to several significant limitations. It relies on GBD study, which, while highly reliable and authoritative, is not without its limitations. Potential biases may arise from data collection methods and statistical methodologies employed. Furthermore, the LSTM model, being a black-box model, presents challenges in interpreting its internal mechanisms, thereby complicating the understanding of how its predictions are derived.

In future research, the integration of diverse data sources – including demographic data, medical records, and environmental monitoring data – holds the potential to yield a more holistic understanding of the factors influencing the burden of NPC, thereby enhancing prediction accuracy. The development of lightweight LSTM models, characterized by fewer parameters and simpler network structures, is anticipated to reduce the complexity and computational cost of model training, facilitating their deployment on mobile and embedded devices. Additionally, investigating interpretability methods for LSTM models, such as attention mechanisms and feature visualization techniques, is expected to deepen the understanding of model predictions and bolster the model’ s transparency and credibility. Furthermore, the integration of NPC burden prediction with related tasks, such as disease diagnosis and treatment option selection, could pave the way for a more comprehensive medical decision support system.

## 5. Conclusion

In conclusion, this study compared the performance of ARIMA, DNN, and LSTM models in predicting the disease burden of nasopharyngeal carcinoma (NPC) in China. Although the DNN model performed well on the training set, ARIMA demonstrated better generalization on the test set. Despite the potential of LSTM, it encountered challenges due to data limitations. The future predictions of all 3 models indicate that although the disease burden of NPC may gradually decrease, it remains extremely severe. Stakeholders can use the model to predict and optimize policies, strengthen interventions, reserve resources, enhance public awareness, and jointly reduce the burden of nasopharyngeal carcinoma disease. Future research should focus on integrating diverse data sources and enhancing model interpretability to improve prediction accuracy and support NPC prevention and treatment strategies.

## Acknowledgments

This article is based on the results of GBD 2021. The authors thank all the epidemiologists, statisticians, and researchers who devoted their time and efforts into the work of GBD 2021.

## Author contributions

**Conceptualization:** Yun Chen, Jian Luo.

**Data curation:** Yun Chen, Kai Zhang, Jian Luo.

**Formal analysis:** Yun Chen, Kai Zhang.

**Funding acquisition:** Yun Chen, Jian Luo.

**Investigation:** Yun Chen, Jian Luo.

**Methodology:** Yun Chen, Jian Luo.

**Project administration:** Jian Luo.

**Resources:** Yun Chen.

**Software:** Yun Chen.

**Supervision:** Jian Luo.

**Validation:** Yun Chen, Kai Zhang.

**Visualization:** Yun Chen, Kai Zhang, Jian Luo.

**Writing – original draft:** Yun Chen, Kai Zhang, Jian Luo.

**Writing – review & editing:** Yun Chen.

## References

[1] Chen YP Chan ATC Le QT Blanchard P Sun Y Ma J. Nasopharyngeal carcinoma. Lancet. 2019;394:64–80. 31178151 10.1016/S0140-6736(19)30956-0

[2] Guo R Mao YP Tang LL Chen L Sun Y Ma J. The evolution of nasopharyngeal carcinoma staging. Br J Radiol. 2019;92:20190244. 31298937 10.1259/bjr.20190244 PMC6774596

[3] Bossi P Gurizzan C Chan A. Immunotherapy for nasopharyngeal carcinoma: the earlier the better. JAMA. 2023;330:1954–5. 38015229 10.1001/jama.2023.22465

[4] Tang LL Chen YP Chen CB. The Chinese society of clinical oncology (CSCO) clinical guidelines for the diagnosis and treatment of nasopharyngeal carcinoma. Cancer Commun (Lond). 2021;41:1195–227. 34699681 10.1002/cac2.12218 PMC8626602

[5] Cui M Cheng H Yuan L. Burden of nasopharyngeal carcinoma in Asia from 1990 to 2021. J Dent. 2025;154:105583. 39880277 10.1016/j.jdent.2025.105583

[6] Lei S Chen L Ji P. Global burdens of nasopharyngeal carcinoma in children and young adults and predictions to 2040. Oral Oncol. 2024;155:106891. 38878356 10.1016/j.oraloncology.2024.106891

[7] Zhang Y Gu S Deng H Shen Z. Global epidemiological profile in nasopharyngeal carcinoma: a prediction study. BMJ Open. 2024;14:e091087. 10.1136/bmjopen-2024-091087 PMC11647315 39658299

[8] Conibear L Reddington CL Silver BJ. Sensitivity of air pollution exposure and disease burden to emission changes in china using machine learning emulation. Geohealth. 2022;6:e2021GH000570. 10.1029/2021GH000570 PMC9207901 35765412

[9] Bhattacharyya N Silver J Bogart M. Profiling disease and economic burden in CRSwNP using machine learning. J Asthma Allergy. 2022;15:1401–12. 36211639 10.2147/JAA.S378469 PMC9532264

[10] Altuhaifa F. Time series prediction of lung cancer death rates on the basis of SEER data. JCO Clin Cancer Inform. 2023;7:e2300011. 37311162 10.1200/CCI.23.00011

[11] Li X Xu C Wang K Yang X Li Y. Data-driven adaptive GM(1,1) time series prediction model for thermal comfort. Int J Biometeorol. 2023;67:1335–44. 37347280 10.1007/s00484-023-02500-9

[12] Na X Han M Ren W Zhong K. Modified BBO-based multivariate time-series prediction system with feature subset selection and model parameter optimization. IEEE Trans Cybern. 2022;52:2163–73. 32639932 10.1109/TCYB.2020.2977375

[13] Wan Y Song P Liu J Xu X Lei X. A hybrid model for hand-foot-mouth disease prediction based on ARIMA-EEMD-LSTM. BMC Infect Dis. 2023;23:879. 38102558 10.1186/s12879-023-08864-y PMC10722819

[14] Benvenuto D Giovanetti M Vassallo L Angeletti S Ciccozzi M. Application of the ARIMA model on the COVID-2019 epidemic dataset. Data Brief. 2020;29:105340. 32181302 10.1016/j.dib.2020.105340 PMC7063124

[15] Claris S Peter N. ARIMA model in predicting of COVID-19 epidemic for the Southern Africa region. Afr J Infect Dis. 2022;17:1–9. 36756487 10.21010/Ajidv17i1.1 PMC9885024

[16] Kriegeskorte N Golan T. Neural network models and deep learning. Curr Biol. 2019;29:R231–6. 30939301 10.1016/j.cub.2019.02.034

[17] Geubbelmans M Rousseau AJ Burzykowski T Valkenborg D. Artificial neural networks and deep learning. Am J Orthod Dentofacial Orthop. 2024;165:248–51. 38302219 10.1016/j.ajodo.2023.11.003

[18] Sinha VB Kudugunta S Sankar AR Chavali ST Balasubramanian VN. DANTE: deep alternations for training neural networks. Neural Netw. 2020;131:127–43. 32771843 10.1016/j.neunet.2020.07.026

[19] Quddus A Shahidi Zandi A Prest L Comeau FJE. Using long short term memory and convolutional neural networks for driver drowsiness detection. Accid Anal Prev. 2021;156:106107. 33848710 10.1016/j.aap.2021.106107

[20] Haralabopoulos G Razis G Anagnostopoulos I. A Modified long short-term memory cell. Int J Neural Syst. 2023;33:2350039. 37300815 10.1142/S0129065723500399

[21] Qin C Chen L Cai Z Liu M Jin L. Long short-term memory with activation on gradient. Neural Netw. 2023;164:135–45. 37149915 10.1016/j.neunet.2023.04.026

[22] Stevens GA Alkema L Black RE; GATHER Working Group. Guidelines for accurate and transparent health estimates reporting: the GATHER statement [published correction appears in PLoS Med. 2016 Aug 9;13(8):e1002116. doi: 10.1371/journal.pmed.1002116.]. PLoS Med. 2016;13:e1002056. 27504831 10.1371/journal.pmed.1002116 PMC4978408

[23] GBD 2021 Diseases and Injuries Collaborators. Global incidence, prevalence, years lived with disability (YLDs), disability-adjusted life-years (DALYs), and healthy life expectancy (HALE) for 371 diseases and injuries in 204 countries and territories and 811 subnational locations, 1990-2021: a systematic analysis for the global burden of disease study 2021. Lancet. 2024;403:2133–61. 38642570 10.1016/S0140-6736(24)00757-8 PMC11122111

[24] Chen X Zheng J Wang J. Global burden and cross-country inequalities in stroke and subtypes attributable to diet from 1990 to 2019. BMC Public Health. 2024;24:1813. 38978043 10.1186/s12889-024-19337-5 PMC11229201

[25] Shi M Yang A Lau ESH. A novel electronic health record-based, machine-learning model to predict severe hypoglycemia leading to hospitalizations in older adults with diabetes: a territory-wide cohort and modeling study. PLoS Med. 2024;21:e1004369. 38607977 10.1371/journal.pmed.1004369 PMC11014435

[26] Tao L Cui Z He Y Yang D. An explainable multiscale LSTM model with wavelet transform and layer-wise relevance propagation for daily streamflow forecasting. Sci Total Environ. 2024;929:172465. 38615782 10.1016/j.scitotenv.2024.172465

[27] Lu S. Research on GDP forecast analysis combining BP neural network and ARIMA model. Comput Intell Neurosci. 2021;2021:1026978. 34804136 10.1155/2021/1026978 PMC8604606

[28] Yang P Cheng P Zhang N Luo D Xu B Zhang H. Statistical machine learning models for prediction of China’s maritime emergency patients in dynamic: ARIMA model, SARIMA model, and dynamic Bayesian network model. Front Public Health. 2024;12:1401161. 39022407 10.3389/fpubh.2024.1401161 PMC11252837

[29] Zhang X Ma R. Forecasting waved daily COVID-19 death count series with a novel combination of segmented Poisson model and ARIMA models. J Appl Stat. 2021;50:2561–74. 37529559 10.1080/02664763.2021.1976119 PMC10388814

[30] Sumi C Ou T Takishima J Shirafuji S. Considerations about L2- and L1-norm regularizations for ultrasound reverberation characteristics imaging and vectoral Doppler measurement. Annu Int Conf IEEE Eng Med Biol Soc. 2022;2022:3882–6. 36085805 10.1109/EMBC48229.2022.9870991

[31] Li Y Li M Zhang L. Evolutionary polynomial regression improved by regularization methods. PLoS One. 2023;18:e0282029. 36800351 10.1371/journal.pone.0282029 PMC9937465

[32] Zhang H He X Yu J He X Guo H Hou Y. L1-L2 norm regularization via forward-backward splitting for fluorescence molecular tomography. Biomed Opt Express. 2021;12:7807–25. 35003868 10.1364/BOE.435932 PMC8713696

[33] Lai M Cao Y Wulff SS Robinson TJ McGuire A Bisha B. A time series based machine learning strategy for wastewater-based forecasting and nowcasting of COVID-19 dynamics. Sci Total Environ. 2023;897:165105. 37392891 10.1016/j.scitotenv.2023.165105

[34] Olsavszky V Dosius M Vladescu C Benecke J. Time series analysis and forecasting with automated machine learning on a national ICD-10 database. Int J Environ Res Public Health. 2020;17:4979. 32664331 10.3390/ijerph17144979 PMC7400312

